# The Induction and Maintenance of Transplant Tolerance Engages Both Regulatory and Anergic CD4^+^ T cells

**DOI:** 10.3389/fimmu.2017.00218

**Published:** 2017-03-06

**Authors:** Alix Besançon, Marije Baas, Tania Goncalves, Fabrice Valette, Herman Waldmann, Lucienne Chatenoud, Sylvaine You

**Affiliations:** ^1^Université Paris Descartes, Sorbonne Paris Cité, Paris, France; ^2^INSERM U1151, Institut Necker-Enfants Malades, Paris, France; ^3^CNRS UMR 8253, Institut Necker-Enfants Malades, Paris, France; ^4^Therapeutic Immunology Group, Sir William Dunn School of Pathology, University of Oxford, Oxford, UK

**Keywords:** transplant tolerance, regulatory T cells, anergy, immunotherapy, CD3 monoclonal antibody

## Abstract

Therapeutic tolerance to self-antigens or foreign antigens is thought to depend on constant vigilance by Foxp3^+^ regulatory T cells (Tregs). Previous work using a pancreatic islet allograft model and a short pulse of CD3 antibody therapy has shown that CD8^+^ T cells become anergic and use TGFβ and coinhibitory signaling as their contribution to the tolerance process. Here, we examine the role of CD4^+^ T cells in tolerization by CD3 antibodies. We show that both Foxp3^+^ Tregs and CD4^+^ T cell anergy play a role in the induction of tolerance and its maintenance. Foxp3^+^ Tregs resisted CD3 antibody-mediated depletion, unlike intragraft Th1 CD4^+^ lymphocytes coexpressing *granzyme B* and *Tbx21*, which were selectively eliminated. Tregs were mandatory for induction of tolerance as their depletion at the time of CD3 antibody therapy or for a short time thereafter, by an antibody to CD25 (PC61), led to graft rejection. Early treatment with CTLA-4 antibody gave the same outcome. In contrast, neither PC61 nor anti-CTLA-4 given late, at day 100 posttransplant, reversed tolerance once established. Ablation of Foxp3 T cells after diphtheria toxin injection in tolerant Foxp3^DTR^ recipient mice provided the same outcome. Alloreactive T cells had been rendered intrinsically unresponsive as total CD4^+^ or Treg-deprived CD4^+^ T cells from tolerant recipients were unable to mount donor-specific IFN-γ responses. In addition, intragraft Treg-deprived CD4^+^ T cells lacked proliferative capacities, expressed high levels of the inhibitory receptor PD-1, and exhibited a CD73^hi^FR4^hi^ phenotype, thus reflecting a state of T cell anergy. We conclude that Tregs play a substantive and critical role in guiding the immune system toward tolerance of the allograft, when induced by CD3 antibody, but are less important for maintenance of the tolerant state, where T cell anergy appears sufficient.

## Introduction

Manipulating the immune system to induce immune tolerance has been the focus of numerous studies in transplantation aiming to promote long-term graft survival and to minimize immunosuppressive drugs with their attendant unwanted side effects. Lessons gathered from experimental models using monoclonal antibodies (Abs), or fusion proteins directed against T cell receptor (TCR) coreceptors (CD4 and CD8) and/or costimulatory molecules (CD28 and CD40L), have unanimously highlighted the central role of CD4^+^Foxp3^+^ regulatory T cells (Tregs) in controlling alloreactive effector T cells (Teff) and thus promoting transplant tolerance and long-term graft survival (1–3). Tregs, operating through mechanisms of linked suppression (tolerance to one set of antigens extended to other antigens coexpressed in the same tissue) and infectious tolerance (generation of new peripherally derived Tregs (pTreg) specific for the original antigen), are thought to be required at both the induction and maintenance phases of tolerance (4–6). Transgenic models of transplantation using CD4 and CD8 Abs have emphasized the induction of Foxp3^+^ pTreg, which can actively and continuously control Teff within the graft microenvironment, creating a form of immune privilege (6–8).

We previously established, in experimental models aiming to reverse autoimmune disease and enable acceptance of transplants, that CD3 Abs possess tolerance promoting properties (9). In particular, we reported that non-Fc-binding CD3 F(ab′)_2_ fragments, administered in a defined therapeutic window, induced long-term survival of fully MHC-mismatched cardiac and pancreatic islet transplantation and implantation of allogeneic stem cells (10–12). Long-term acceptance of second grafts from the original, but not third-party donors, proved that antigen-specific tolerance had been induced (10). We have previously shown that alloreactive CD8^+^ T cells become anergic within anti-CD3 tolerized mice and use TGFβ and coinhibitory signaling as their contribution to the tolerance process (13). Our previous work revealed that Foxp3^+^ Tregs accumulated within the grafts of CD3 Ab-treated mice. However, the role of Tregs in the induction and/or the longevity of tolerance was not investigated. Here, we now show that CD4^+^Foxp3^+^ Tregs, spared from depletion by CD3 Ab, are crucial for the induction of transplant tolerance, whereas other alloantigen-specific CD4^+^ T cells become anergic, as was the case for CD8^+^ T cells (13). We conclude that Treg may be relatively dispensable for tolerance maintenance, which predominantly manifests itself as T cell anergy.

## Materials and Methods

### Mice

C57BL/6, RAG^−/−^ C57BL/6, and BALB/c mice were bred in our facility under specific pathogen-free conditions. Foxp3^DTR^ mice were a gift from D.A. Gross (INSERM U1151, Paris). Blood glucose was measured using ACCU-CHECK Performa glucometer (Roche Diagnostics). Experiments were conducted according to European Directives (2010/63/UE) and were approved by the Ethical Committee of Paris Descartes University (#14-075) and the French Ministry of Education and Research (#04462.02).

### Pancreatic Islet Isolation and Transplantation

Pancreatic islets were separated by density gradient centrifugation (Histopaque, Sigma-Aldrich) after *in situ* digestion with collagenase P (Roche Applied Science) and transplanted (300 islets) under the kidney capsule of diabetic recipients. Diabetes was induced by a single injection of streptozotocin (Sigma-Aldrich) at 225 mg/kg. Diagnosis of graft rejection was made after three glucose measurements >250 mg/dl.

### Abs and *In Vivo* Treatments

The cell line SP2/0 producing the genetically engineered 145-2C11 F(ab′)_2_ fragments (14) as well as the CTLA-4 Ab (4F10) were provided by J. A. Bluestone (UCSF, San Francisco, CA, USA). CD3 F(ab′)_2_ fragments were injected i.v. at the dose of 50 μg/day for 5 days, starting on day 7 posttransplant as previously described (10). Purified anti-CTLA-4 Abs were injected i.p. at the dose of 500 μg/day. Purified CD25 Abs (PC61, Bio X Cell) were administered at the dose of 300 μg i.p. For flow cytometry, CD4 (GK1.5), CD8 (53-6.7), TCR Vβ (H57-597), CD44 (IM7), CD62L (MEL-14), CD69 (H1.2F3), CD122 (TM-β1), CTLA-4 (4F10-11), and Ki67 (B56) Abs were purchased from BD Biosciences and Foxp3 (FJK-16S), FR4 (ebio12A5), CD73 (ebioTY/11.8) and PD-1 (RMP1-30) Abs purchased from eBioscience.

Diphtheria toxin (DT) (gift from D. A. Gross, INSERM U1151, Paris) was injected i.p. at the dose of 25 μg/kg for two consecutive days in transplanted Foxp3^DTR^ recipient mice showing established tolerance after CD3 Ab therapy.

### IFN-γ ELISPOT

The method has been previously described (15). Briefly, PVDF plates were coated with anti-IFN-γ Ab (U-Cytech). Purified CD4^+^ T cells (10^5^/well) were incubated with 10^5^ irradiated splenocytes from C57BL/6, BALB/c, or C3H mice. After a 20-h culture, IFN-γ was detected using biotinylated anti-IFN-γ Ab, streptavidin-horseradish peroxidase, and Sigma*FAST* NBT-BCIP (Sigma-Aldrich). Results were expressed as spot-forming units/10^6^ cells.

### *In Vitro* Suppression Assays

Effector T cells were obtained by immunizing C57BL/6 mice with BALB/c antigens (30 × 10^6^ spleen cells i.p. on day 20 and day 10). T cells were isolated by magnetic sorting (T cell isolation kit, Miltenyi Biotec), labeled with the violet proliferation dye (VPD450), and coincubated at a 1/1 ratio with CD4^+^CD25^+^ Tregs (5 × 10^4^ cells/well) isolated from transplanted recipients (treated or not with CD3 Abs). Cells were stimulated with T cell-depleted irradiated splenocytes from BALB/c mice for 5 days. Proliferation was evaluated by flow cytometry by gating on CD8^+^ Teff among living cells. In parallel experiments, cells were stimulated with mitogenic CD3 Abs (145-2C11, 2 μg/ml). When needed, CTLA-4 Abs were added at the dose of 20 μg/ml on day 0.

### Single-Cell PCR

Individual CD4^+^ T cells were FACS sorted from the spleen or the islet allografts of untreated or CD3 Ab-treated recipients. After cell lysis by heating/cooling steps, RNA was specifically retrotranscribed using MuLV Reverse Transcriptase (Applied Biosystems) and 3′ specific primers (Eurofins MWG). The resulting cDNA was next amplified (first PCR with all primers). Product of this first PCR was then subjected to a second PCR using SYBR Green PCR Master Mix (Applied Biosystems) for each primer pairs. To ensure that each well contained a T cell, *Cd3e* mRNA was amplified simultaneously with the genes of interest. HPRT was the housekeeping gene. Multiplex single-cell PCR was performed for the following genes: granzymes A and B (*Gzma, Gzmb*), perforin-1 (*Prf1*), and FasLigand (*Fasl)*, which provide killing abilities; the transcription factors T-bet (*Tbx21*) and the β chain of the IL-12 receptor (*Il-12rb*), which promote IFN-γ expression; and the killer cell lectin-like receptor G-1 (*Klrg1*), which marks terminally differentiated Teff.

### Statistics

Cumulative actuarial graft survival was calculated using the Kaplan–Meier method. The statistical comparison was performed using the log-rank (Mantel-Cox) test. When appropriate, Mann–Whitney, Student’s *t*-test (paired or unpaired), or Chi square (χ^2^) test was used. A *p* value <0.05 was considered significant.

## Results

### Foxp3^+^ Tregs Resist CD3 Ab-Induced Depletion and Are Mandatory for Tolerance Induction

**Figure 1:**
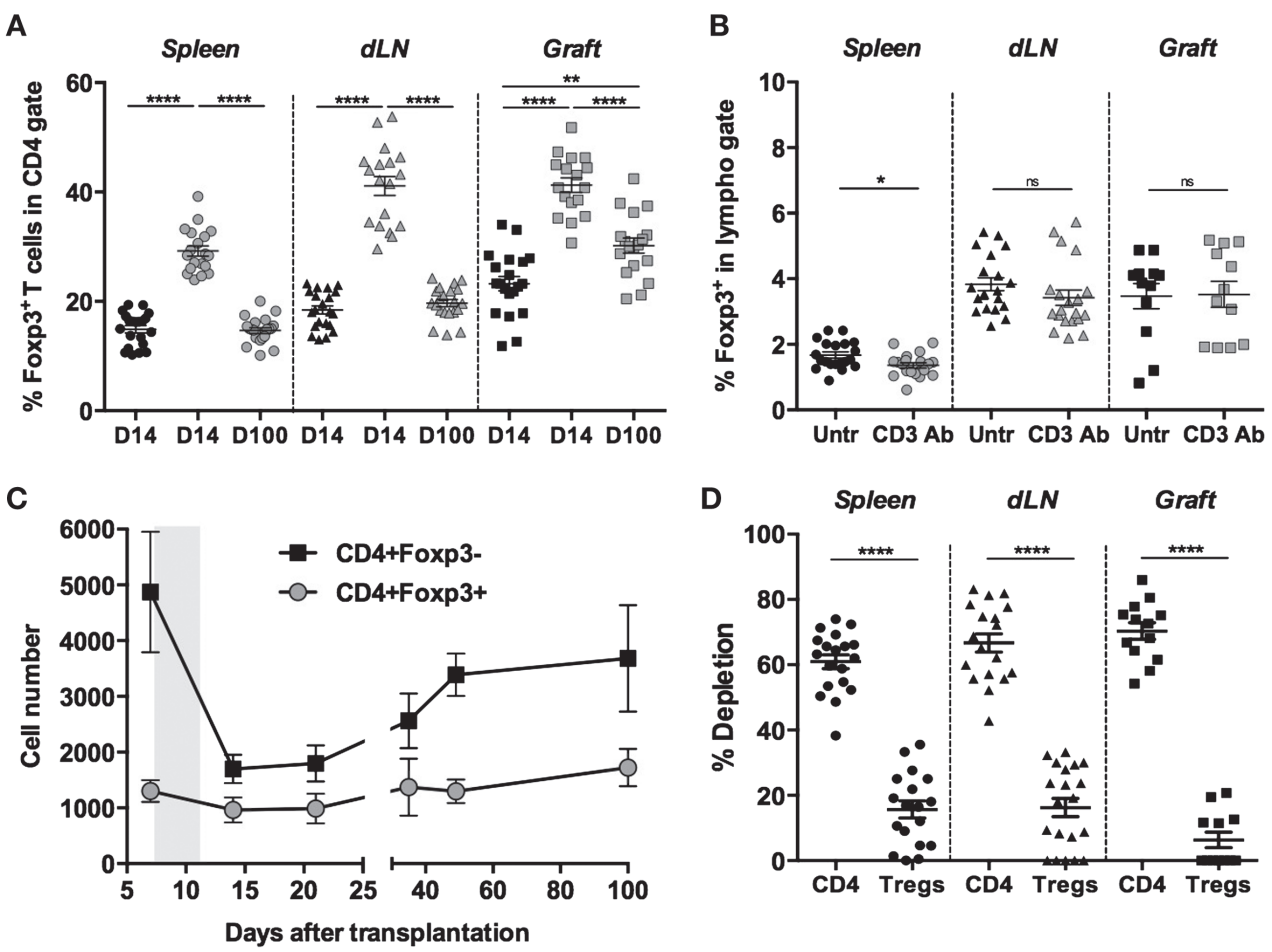
**Foxp3^+^ regulatory T cells (Tregs) resist CD3 antibody (Ab)-induced depletion**. Spleen, draining renal lymph nodes (dLN), and islet allografts were recovered on day 14 or 100 after transplantation from C57BL/6 mice treated or not with CD3 Abs (treatment at day 7 posttransplant for 5 consecutive days). Foxp3 expression was evaluated in the CD4^+^ T cell population **(A)** and in the total lymphoid gate **(B)** (black symbols: untreated; gray symbols: CD3 Ab-treated). **(C)** Number of Foxp3^+^ regulatory and Foxp3^−^ effector CD4^+^ T cells present within the islet allograft before and after CD3 Ab therapy (gray area). **(D)** Percentage of cell depletion evaluated in CD4^+^Fox3^−^ T cells or in CD4^+^Foxp3^+^ T cells after treatment with CD3 Abs (*p < 0.02, **p < 0.002, and ****p < 0.0001).

We previously reported that short-course treatment with CD3 Ab F(ab′)_2_ fragments induced long-term survival and immune tolerance toward fully mismatched pancreatic islets (10). Here, we investigated the impact of CD3 Ab treatment on Foxp3^+^ Tregs and their role in tolerance induction. C57BL/6 mice, rendered diabetic after one injection of streptozotocin, were transplanted with pancreatic islets from BALB/c mice and were treated on day 7 and for 5 consecutive days with CD3 Ab F(ab′)_2_. Analysis of CD4^+^ T cells on day 14 posttransplant revealed after CD3 Ab treatment a significant increase in the proportion of CD4^+^Foxp3^+^ T cells, not only in secondary lymphoid organs [spleen: 14.9 ± 0.7 vs 29.2 ± 0.9%, draining renal lymph nodes (dLN): 18.4 ± 0.8 vs 41.1 ± 1.7%] but also in the islet allografts (23.2 ± 1.3 vs 41.2 ± 1.6%) (Figure 1A). However, this was not due to a substantial systemic expansion of Tregs since the frequency of Foxp3^+^ T cells within the total lymphoid population was not significantly modified after CD3 Ab therapy (Figure 1B). Indeed, CD3 Ab preferentially targeted CD4^+^Foxp3^−^ T cells as shown by their drastic decreased number detected after treatment in the transplanted islets where approximately 70% of them were depleted as compared to a mean of 8.5% for CD4^+^Foxp3^+^ Tregs (Figures 1C,D). On day 100 posttransplant, Foxp3^+^Treg frequencies were back to normal in the spleen and dLN, while they were still high within the graft (30.2 ± 1.4%) (Figure 1A).

**Figure 2:**
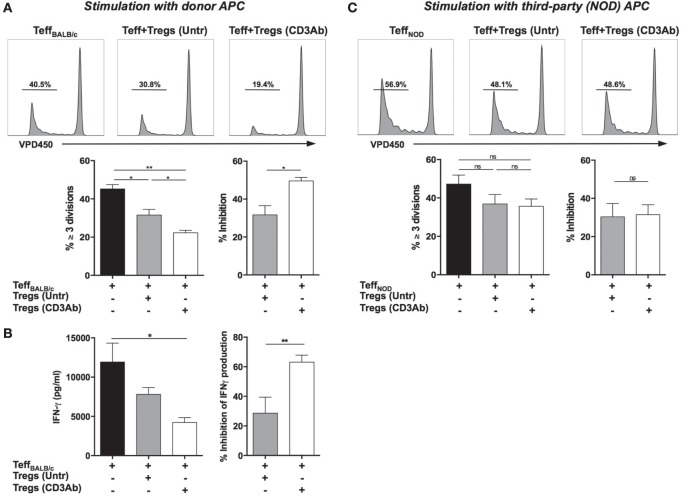
**Foxp3^+^ regulatory T cells (Tregs) exhibit antigen-specific suppressive capacities**. **(A,B)** Co-cultures were established by incubating VPD450-labeled effector CD8^+^ T cells (Teff) from C57BL/6 mice immunized with BALB/c antigens with CD4^+^CD25^+^ T cells Tregs isolated from transplanted mice treated or not with CD3 antibodies (Abs). Cells were plated at a 1/1 ratio and stimulated with T cell-depleted irradiated BALB/c splenocytes (APC) for 5 days. **(A)** Representative histograms of Teff_BALB/c_ proliferation are shown (upper panels) as well as the proportion of Teff_BALB/c_ that divided three times or more and the percentage of inhibition of dividing cells (≥3 divisions) in the presence of Tregs (lower panels) (*n* = 6, *p < 0.04, **p < 0.008). **(B)** Supernatants were collected after 72 h of coculture, and the production of IFN-γ by Teff_BALB/c_ in the presence or absence of Tregs was measured by ELISA (*n* = 6, *p* < 0.03). Percentage of inhibition of IFN-γ production by Tregs is also represented (**p < 0.01). **(C)** Teff were recovered from C57BL/6 mice previously immunized with third-party antigens (spleen cells from NOD mice), labeled with VPD450, and cocultured at a 1:1 ratio with Tregs from untreated or CD3 Ab-treated recipients in the presence of T cell-depleted splenocytes from NOD mice. Upper panel: Representative histograms of CD8^+^ Teff_NOD_ proliferation as measured by VPD450 dilution in the presence or absence of Tregs. Lower panel: Proportion of VPD450-labeled Teff_NOD_ that underwent three divisions or more (*n* = 6) and percentage of inhibition of Teff_NOD_ proliferation cultured with Tregs.

The *in vitro* ability of Tregs to inhibit the proliferation of VPD450-labeled Teff (recovered from C57BL/6 mice immunized with BALB/c antigens) stimulated with irradiated BALB/c APCs was evaluated. Tregs from CD3 Ab-treated mice showed a higher efficacy in inhibiting donor-specific proliferative responses as compared to Tregs from untreated recipients: 49.5 vs 31.7% inhibition, respectively (Figure 2A). Similarly, IFN-γ production by alloreactive T cells was significantly inhibited by Treg from CD3 Ab-treated recipients (Figure 2B). Such an outcome was not observed when Tregs were cocultured with VPD450-labeled Teff recovered from C57BL/6 mice immunized with third-party (NOD) antigens and stimulated by irradiated NOD APCs (Figure 2C). In this situation, Tregs from untreated and CD3 Ab-treated mice showed modest yet similar suppressive capacities.

**Figure 3:**
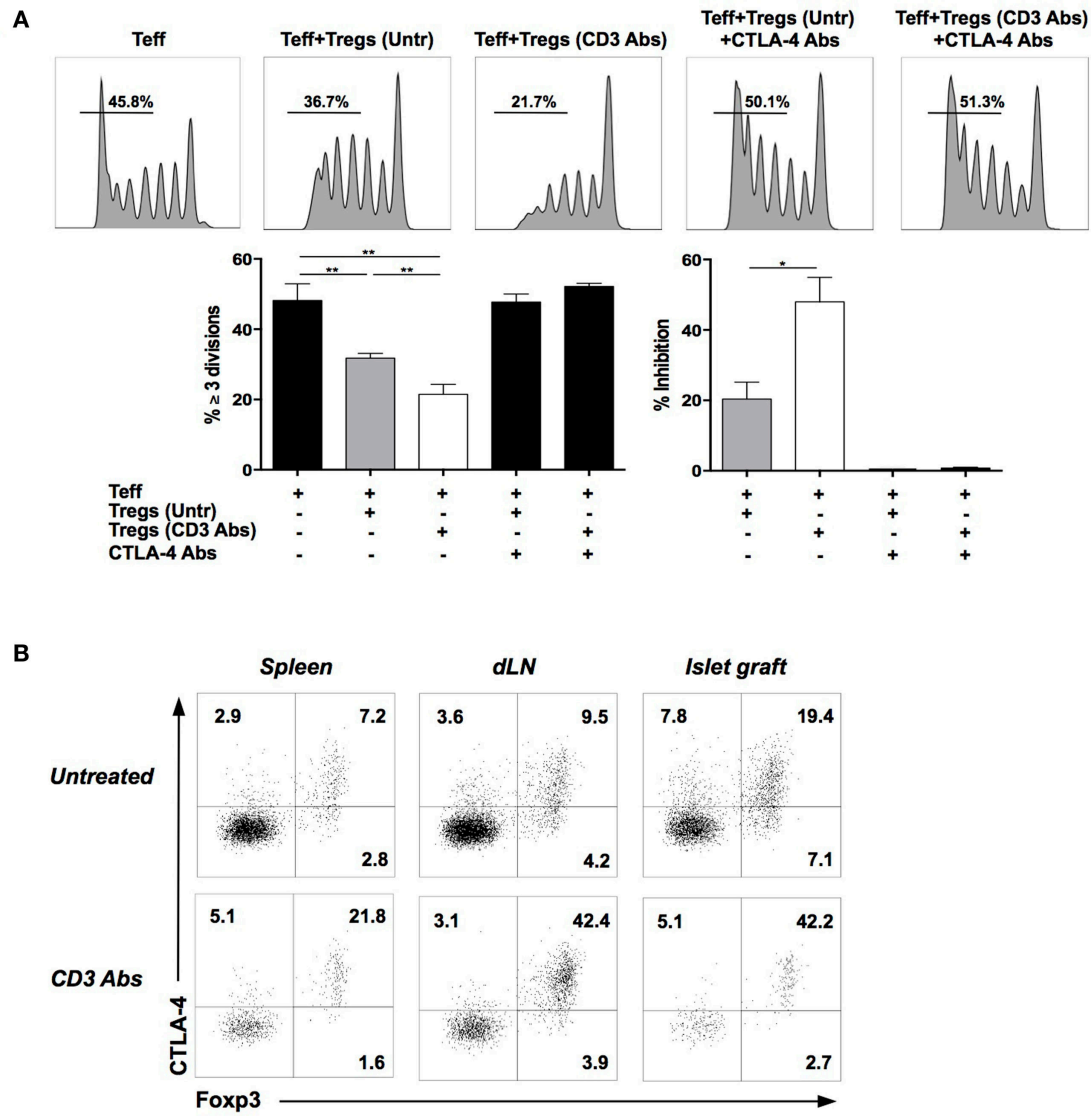
**Regulatory T cells (Tregs) involved in tolerance induction express and require CTLA-4 to manifest their suppressive function**. **(A)** *In vitro* suppressive capacity of Tregs is CTLA-4 dependent. CD4^+^CD25^+^ Tregs were isolated from C57BL/6 mice transplanted with BALB/c islets and treated or not with CD3 antibody (Ab). Effector T cells (Teff) were recovered from C57BL/6 mice previously immunized with BALB/c antigens. Teff and Tregs were coincubated at a 1:1 ratio and stimulated with CD3 Abs (145-2C11) for 5 days. Neutralizing CTLA-4 Abs were added to the culture on day 0. Representative histograms of CD8^+^ Teff proliferation are shown (upper panels) as well as the proportion of CD8^+^ Teff that divided three times or more and the percentage of inhibition of dividing cells (≥ 3 divisions) in the presence of Tregs (lower panels) (*p < 0.04, **p < 0.008). **(B)** Representative dot plots of Foxp3 and CTLA-4 coexpression by spleen, draining lymph nodes, or graft-infiltrating CD4^+^ T cells on day 14 after pancreatic islet transplantation and CD3 Ab therapy (*n* = 6–12).

Regulatory T cells exerted their suppressive effect in a CTLA-4-dependent manner as addition of CTLA-4 binding Abs completely reversed the inhibition of VPD450-labeled Teff proliferation (induced by CD3 Abs) (Figure 3A). Of note, expression of CTLA-4 after CD3 Ab treatment was almost exclusively restricted to Foxp3^+^ Tregs (Figure 3B).

**Figure 4:**
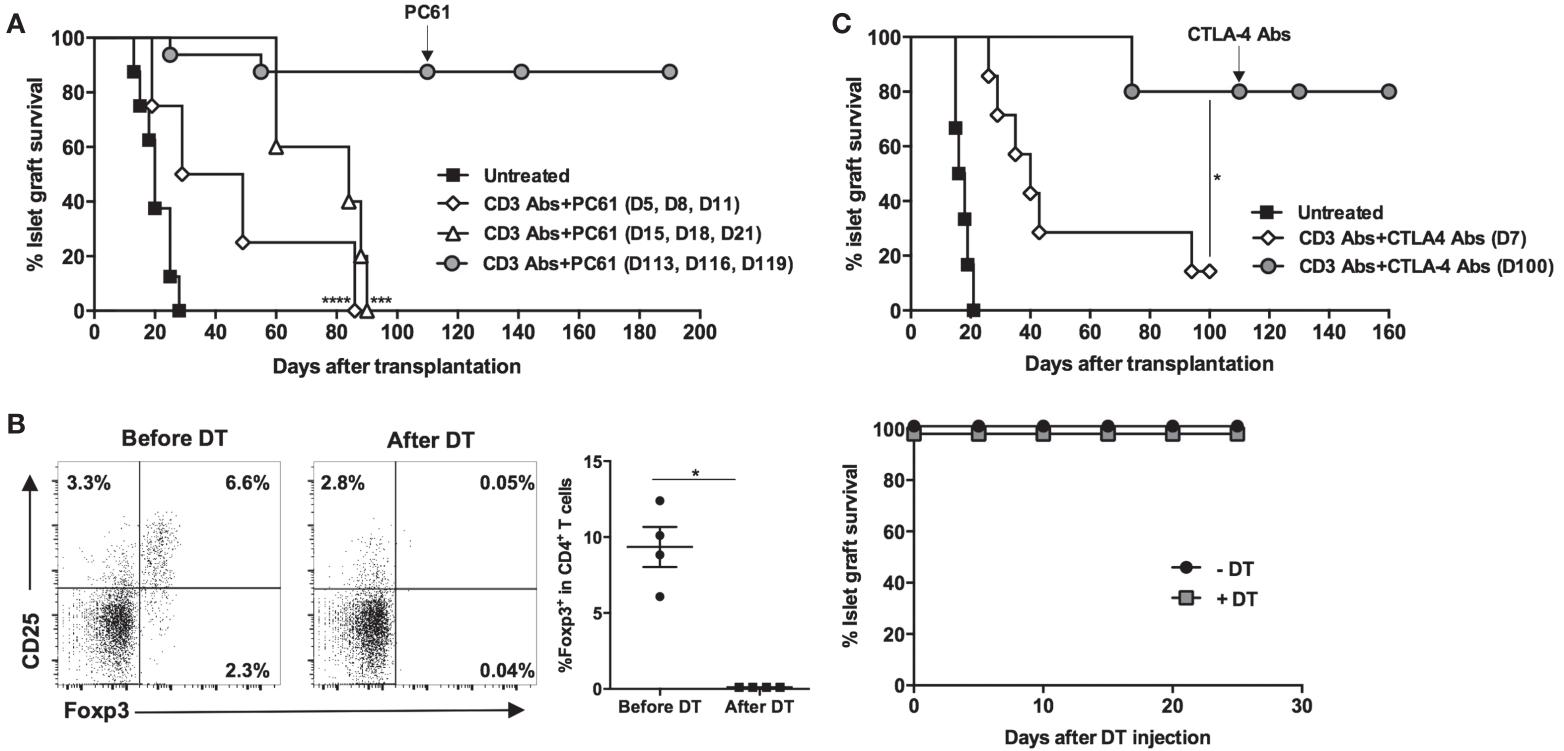
**Induction and maintenance of transplant tolerance engage distinct regulatory mechanisms**. C57BL/6 mice were transplanted with BALB/c islets and treated with CD3 antibodies (Abs) F(ab′)_2_. **(A)** Regulatory T cell (Tregs)-depleting CD25 Ab (PC61) was administered at the dose of 300 μg i.p. at different time points: on days 5, 8, and 11 (at the time of CD3 Ab therapy, *n* = 4), on days 15, 18, and 21 (at the time of T cell reconstitution, *n* = 5), or on days 113, 116, and 119 posttransplant once tolerance was established (*n* = 5) (***p = 0.0004 and ****p < 0.0001 when comparing CD3 Ab− to CD3 Ab+ PC61-treated mice). **(B)** Foxp3^DTR^ mice were transplanted with BALB/c islets and treated on day +7 with CD3 Abs. Tolerant mice were injected (*n* = 4) or not (*n* = 3) with diphtheria toxin (DT) at the dose of 25 μg/kg for 2 consecutive days. Treg elimination was analyzed in the blood 48 h after DT injection (*p = 0.027) (left panels), and islet graft survival was monitored (right panel). **(C)** CTLA-4 Ab was injected at the dose of 500 μg, five injections 3 days apart, starting either on day 7 (*n* = 8) or day 110 posttransplant (*n* = 5) (*p < 0.027 between CD3 Ab and CD3 + CTLA-4 Ab-treated mice).

To further investigate the respective role of Foxp3^+^ Treg-mediated suppression, we performed three series of *in vivo* experiments using CD25 Ab (PC61), which preferentially depletes Foxp3^+^ Tregs. Administration of PC61 at the time of CD3 Ab therapy, or 10 days after the end of treatment (when T cell compartment was actively reconstituting), abrogated permanent graft survival and tolerance induction (Figure 4A). In contrast, injection of PC61 on day 110, once tolerance was established, did not precipitate graft rejection (Figure 4A). To confirm this last result, Foxp3^DTR^ recipient mice were transplanted with BALB/c islets, treated with CD3 Abs, and, once tolerant, were injected with DT. Although Treg ablation was complete, islet allografts survived in treated mice (Figure 4B). *In vivo* neutralization of CTLA-4 provided identical results to the one obtained with PC61, i.e., inhibition of tolerance when CTLA-4 Ab was administered early at the time of CD3 Ab treatment but not when given late at day 100 (Figure 4C).

### CD3 Ab Therapy Abrogates Anti-Donor Th1 Responses and Induces Anergy

**Figure 5:**
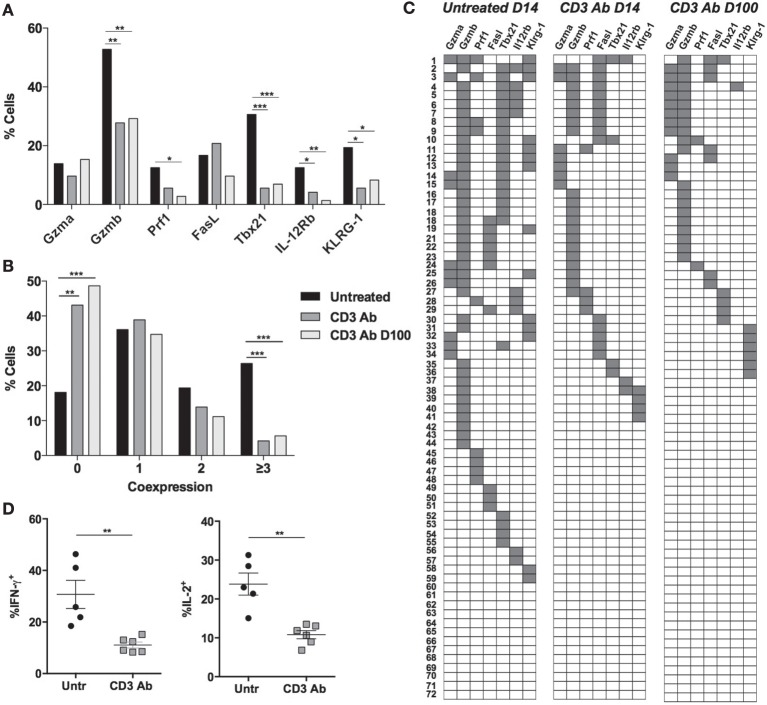
**Long-term downregulated coexpression of effector genes by individual graft-infiltrating CD4^+^ T cells after CD3 antibody (Ab) therapy**. C57BL/6 mice were transplanted under the kidney capsule with BALB/c pancreatic islets and treated or not with CD3 Ab F(ab′)_2_ fragments (50 μg/day for 5 days, starting on day 7 posttransplant). Individual CD4^+^ T cells (*n* = 72) present within the graft were FACS sorted on day +14 or day +100 posttransplant and subjected to multiplex gene expression analysis. **(A)** Proportion of CD4^+^ T cells among the 72 cells tested that expressed *Gzma, Gzmb, Prf1, Fasl, Tbx21, Il-12rb*, and *Klrg1* mRNA in each group. **(B)** Multifunctionality distribution of intragraft CD4^+^ T cells. **(C)** Coexpression of inflammatory and cytotoxic molecules by individual graft-infiltrating CD4^+^ T cells. Each row represents one individual cell that is numbered. Each column represents a different gene. For better visualization of coexpression patterns, individual cells were ordered by the degree of gene coexpression (Figures 1A,B: χ^2^ test, *p < 0.05, **p < 0.007, ***p = 0.0007, ***p < 0.0001). **(D)** Intragraft CD4^+^ T cells were analyzed on day 14 for their capacity to produce IFN-γ and IL-2 following PMA/ionomycin stimulation for 4 h (5–6/group) (**p = 0.0043).

Multiplex single-cell PCR was used to approach the gene signature of individual alloreactive CD4^+^ T cells in response to CD3 Abs. On day 14 posttransplant, we sorted individual CD4^+^ T cells from the islet allografts (72 cells) or spleen (48 cells) of 3 distinct recipients, treated or not with CD3 Ab, and we analyzed the coexpression of Th1 inflammatory and cytotoxic genes (Figure 5). In untreated mice, half of graft-infiltrating CD4^+^ T cells expressed *Gzmb* and 31% expressed the transcription factor *Tbx21* (T-bet), the master regulator of Th1 differentiation (Figure 5A). Of note, most *Tbx21^+^*CD4^+^ T cells coexpressed *Gzmb* (Figure 5C). *Klrg1*, which marks differentiated end-stage T cells, and *Il-12rb* were detected within the *Tbx21^+^Gzmb*^+^CD4^+^ T cell subpopulation. *Fasl* was detected in a fraction of cells and was associated, at least in part, with *Gzmb*. More than 25% of intragraft CD4^+^ T cells coexpressed three or more of the seven genes tested (Figure 5B).

CD3 Ab treatment eliminated the *Gzmb^+^Tbx21^+^*CD4^+^ T subpopulation from the transplanted tissue (Figure 5C). Expression of *Tbx21* was greatly reduced (from 30.6 to 5.6%) as well as that of *Gzmb, Il-12rb*, and *Klrg1* (Figure 5A). Some cells still coexpressed *Fasl* and *Gzmb* (Figure 5C). The frequency of cells negative for all selected genes increased from 18.1% in untreated mice to 43.1% in CD3 Ab-treated mice where less than 5% of graft-infiltrating CD4^+^ T cells coexpressed three to four genes (Figure 5B). At the protein level, this translated into a significant decreased ability of intragraft CD4^+^ T cells to produce IFN-γ and IL-2 after CD3 Ab therapy (Figure 5D). Multiplex single-cell PCR was also performed on day +100 posttransplant and confirmed the downregulated gene expression profile of graft-infiltrating CD4^+^ T cells (Figures 5A–C).

**Figure 6:**
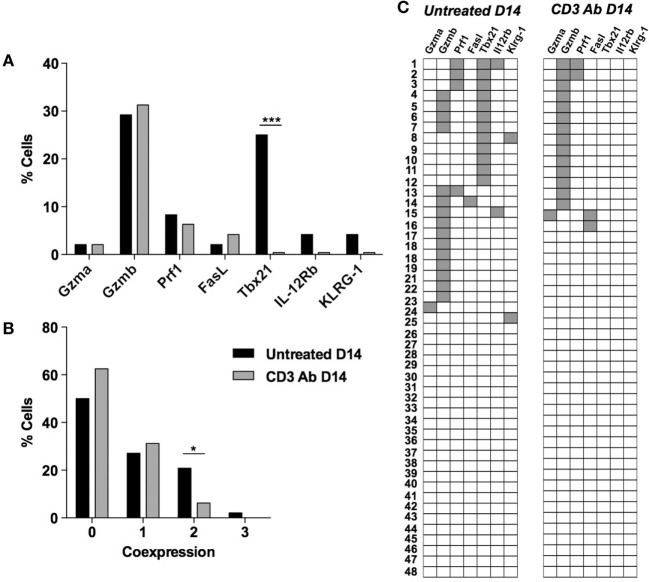
**Multiplex gene expression analysis on individual spleen CD4^+^ T cells after CD3 antibody (Ab) therapy**. Individual CD4^+^ T cells (*n* = 48) were sorted on day +14 from the spleen of C57BL/6 mice transplanted with BALB/c islets and treated or not CD3 Ab on day +7 posttransplant. Expression of *Gzma, Gzmb, Prf1, Fasl, Tbx21, Il-12rb, and Klrg1* mRNA was measured in each cell. Results show the proportion of splenic CD4^+^ T cells positive for each gene **(A)**, the proportion of splenic CD4^+^ T cells coexpressing zero to three of the selected genes **(B)** and the coexpression pattern **(C)** (Figures 3A,B: χ^2^ test *p = 0,037, ***p = 0.0002).

In the spleen, *Gzmb* and *Tbx21* were also expressed by CD4^+^ T cells recovered from untreated recipients but were not systematically coexpressed by the same cells (Figures 6A–C). *Tbx21* expression was completely abrogated after CD3 Ab treatment.

**Figure 7:**
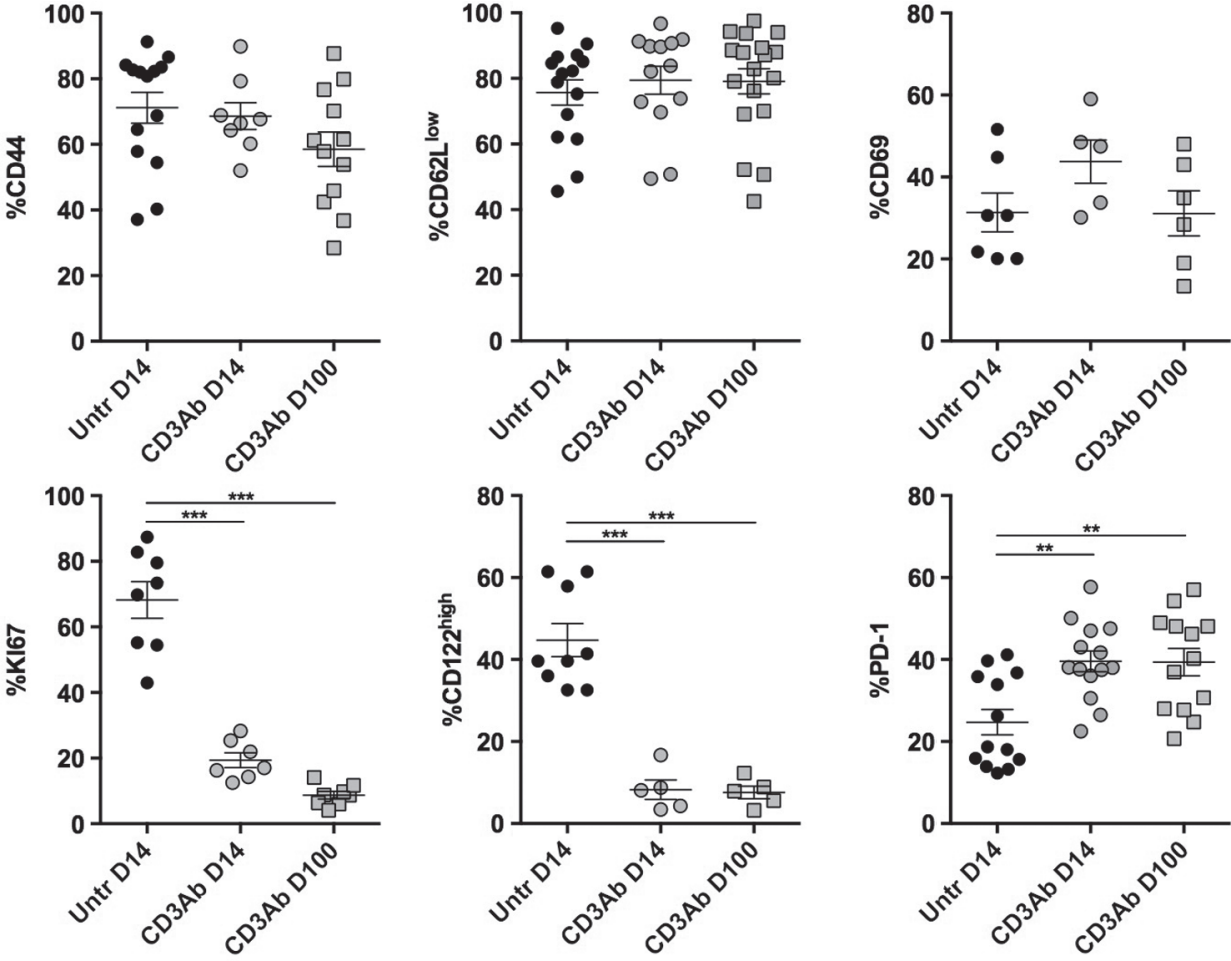
**Phenotypic and functional characteristics of intragraft CD4^+^ T cells after CD3 antibodies (Abs) therapy**. On day +14 posttransplant, pancreatic islet allografts were recovered from C57BL/6 mice treated or not with CD3 Abs (black circles: untreated; gray squares: CD3 Ab treated). Intragraft CD4^+^ T cells were analyzed for their expression of CD44, CD69, CD122, Ki67, and PD-1 (5–15/group) (*p < 0.05, **p < 0.005, ***p < 0.0005).

The phenotypic analysis confirmed the hyporesponsive profile of graft-infiltrating CD4^+^ T cells after CD3 Ab therapy. On day 14 and up to day 100 posttransplant, although they expressed an antigen-experienced CD44^high^CD62L^low^CD69^+^ phenotype, they continually displayed very low expression of the proliferation marker Ki67 and CD122 (β chain of IL-2 and IL-15 receptors) but high expression of inhibitory receptor PD-1 (Figure 7).

**Figure 8:**
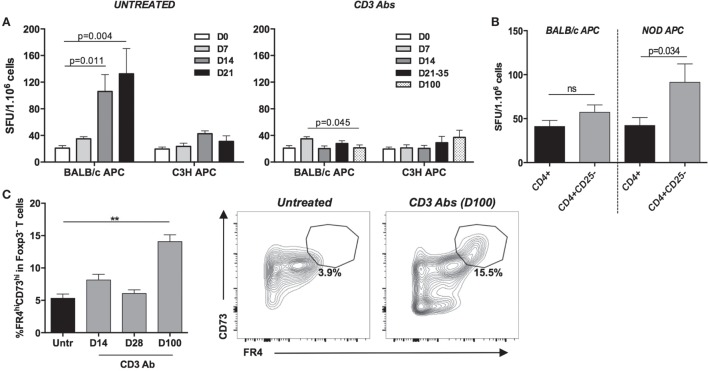
**Assessment of CD4^+^ T cell anergy in tolerant hosts**. **(A)** CD4^+^ T cells from CD3 antibody (Ab)-treated mice display an impaired antidonor reactivity. Spleens were harvested at various time points from C57BL/6 mice transplanted with BALB/c islets and treated or not with CD3 Abs F(ab′)_2_ fragments. CD4^+^ T cells were purified, and production of IFN-γ was measured by Elispot after a 24-h incubation with irradiated T cell-depleted splenocytes (APC) from BALB/c or C3H donors (4–8 mice/group/time point). **(B)** Antidonor responses of Treg-deprived CD4^+^CD25^−^ T cells isolated from tolerant CD3 Ab-treated mice (day 100) were evaluated by IFN-γ Elispot assay using irradiated BALB/c or third-party (NOD) APCs (*n* = 5/6) (*p = 0.034). **(C)** Expression of CD73 and FR4 was analyzed on graft-infiltrating CD4^+^Fox3^−^ T cells recovered from untreated (day 14, *n* = 5) or CD3 Ab-treated recipients (day 14, *n* = 4; day 28, *n* = 4; day 100, *n* = 8). The mean proportion of CD4^+^Fox3^−^ T cells coexpressing high levels of CD73 and FR4 at day 100 (left panel) is shown as well as representative dot plots of CD73 and FR4 coexpression pattern (right panel) (**p < 0.009).

Functional assays (IFN-γ Elispot) were performed to assess the donor-specific reactivity of CD4^+^ T cells at different time points after transplantation and treatment. CD4^+^ T cells from CD3 Ab-treated mice were unable to mount an efficient IFN-γ response toward BALB/c antigens up to day 100 posttransplant, well after the Ab had cleared from the blood circulation and T cells were back to their normal levels (Figure 8A). In contrast, CD4^+^ T cells from untreated recipients displayed an antigen-specific increase of IFN-γ production on day 14 and 21 after transplantation, i.e., at the time of rejection.

Analysis of tolerant C57BL/6 recipients (day 100) further showed that Treg-depleted CD4^+^ T cells (CD4^+^CD25^−^) were hyporesponsive to donor antigens similar to that of total CD4^+^ T cells (Figure 8B). In contrast, CD4^+^ T cell responses toward third-party antigens increased in the absence of Tregs. Finally, we analyzed the coexpression of CD73 and FR4 on graft-infiltrating CD4^+^Foxp3^−^ T cells, two markers that have been shown to discriminate naïve, effector/memory, and anergic CD4^+^ T cells (16–18). In CD3 Ab-treated tolerant mice, at day 100 posttransplant (but not at earlier time points, e.g., day 14 and 28), the CD4^+^Foxp3^−^ population was highly enriched in CD73^hi^FR4^hi^ T cells (14 vs 6.5% in untreated mice), so identifying likely anergic T cells (Figure 8C).

## Discussion

A large body of experimental work in autoimmunity and transplantation has implicated Foxp3^+^ Tregs in orchestrating and sustaining dominant mechanisms of tolerance, allowing a constant control of Teff. Data presented in this article support the fact that transplant tolerance relies on cell extrinsic and intrinsic mechanisms engaging both FoxP3^+^ Tregs and CD4^+^ T cell anergy in a timely manner. By using a fully mismatched islet allograft model and CD3 Ab therapy, we demonstrated that CTLA-4^+^Foxp3^+^ Tregs resist depletion by the CD3 Ab, unlike effector alloreactive *Gzmb^+^Tbx21^+^*CD4^+^ Th1 T cells, so enabling them to orchestrate the subsequent tolerance inducing events. Even though FoxP3^+^ Treg could be found in graft infiltrates indefinitely, treatment of hosts maintaining their grafts long term with the PC61 and CTLA-4 Abs did not break the tolerant state. Detailed analysis of graft-infiltrating T cells was consistent with the alloreactive members being anergic.

Our previous work demonstrated the capacity of CD3 Ab to induce transplant tolerance (10–12). The therapeutic window was of crucial importance, and transplant tolerance was only obtained when the treatment was applied at the time of Teff priming to the alloantigens. One direct effect of the Ab was a partial and transient depletion of CD4^+^ and CD8^+^ T cells in secondary lymphoid organs and in the grafts. Importantly, all T cell subsets were not equally sensitive to the CD3 Ab-depleting effect. Single-cell multiplex PCR, optimized to discriminate T cell heterogeneity and functional behavior (19, 20), showed that CD3 Ab preferentially targeted and eliminated CD4^+^ T cells coexpressing *Gzmb/Tbx21*. This finding is reminiscent of our previous report showing that cytotoxic intragraft CD8^+^ T cells coexpressing granzyme B and perforin were selectively deleted (13). Apoptosis of these alloreactive T cells by CD3 Abs was mandatory for long-term graft survival and was dependent on the Fas/FasLigand pathway: *in vivo* FasL blockade abrogated T cell elimination and CD3 Ab-induced tolerance to islet transplant (13). In this study, we demonstrated that *in vivo*, in contrast to Teff, Foxp3^+^ Tregs were resistant to CD3 Ab-mediated depletion. Consequently, their proportion increased in both lymphoid organs and the transplanted islets at the end of treatment. The molecular mechanisms underlying such Treg resistance to apoptosis are not fully understood, but may include a reduced expression and activation of signaling molecules downstream of the TCR/CD3 complex as well as a decreased expression of the CD3 molecules at the cell surface compared to CD4^+^Foxp3^−^ T lymphocytes (21–23).

Regulatory T cells are mandatory for the induction of tolerance as their depletion at the time of CD3 Ab therapy, or for a short time thereafter, abolished long-term graft survival demonstrating a critical role of Tregs in that early period when the immune system is responding to graft alloantigens. At this time, numerically dominant Tregs may influence residual and reconstituting naïve T cells inhibiting their differentiation into Th1 effectors. During this phase, Tregs from CD3 Ab-treated mice become endowed with potent antigen-specific suppressive properties as they appeared to inhibit proliferation and IFN-γ secretion of effector cells stimulated with donor antigens more efficiently than Tregs from untreated recipients, or effector cells specific for third-party antigens. CTLA-4 was necessary for Treg-suppressive functions as CTLA-4 blockade completely abrogated their inhibitory effect *in vitro* and prevented CD3 Ab-induced tolerance and long-term survival *in vivo*.

Our results are in accordance with many experimental studies reporting that Treg depletion (mainly by using the PC61 Ab) around the time of grafting prevented the induction of transplant tolerance (Table S1 in Supplementary Material). However, very few have addressed the impact of Treg elimination once tolerance has been established. Surprisingly, in our model, tolerance was sustained after administration of PC61 or CTLA-4 Abs on day >100 posttransplant, thereby suggesting that long-term maintenance of tolerance was not solely dependent on CTLA-4^+^Foxp3^+^ Treg activity. Survival of islet allografts in Foxp3^DTR^ mice after DT injection further supported this point. It was possible that other types of regulatory T-cells were operating within the graft. For example, IL-10-producing T-cells have been implicated in some forms of therapeutic tolerance (24–26). We have excluded this by demonstrating that CD3 Ab therapy can tolerize even in IL-10 knockout mice (unpublished data). It may also be possible that access of long-term tolerated grafts to the inhibitory effects of Ab may not be equivalent in the inductive and long-term maintenance phases of tolerance.

However, we have provided evidence supporting the notion that tolerance maintenance is associated with the persistence of CD4^+^ anergic T cells. First, single-cell multiplex PCR showed that, following CD3 Ab-induced apoptosis of alloreactive *Gzmb^+^Tbx21^+^* Th1 cells, most of the remaining CD4^+^ T cells expressed none or only one of the selected inflammatory genes over long term, thereby highlighting the absence of potent effector activity in tolerated grafts. Second, in accordance with PCR data, intragraft CD4^+^ T cells show weak proliferation capacities as revealed by the drastically reduced expression of the proliferation marker Ki67 and of the β subunit of the IL-2 and IL-15 receptors (CD122) up to day +100 posttransplant. Third, we demonstrated that total CD4^+^ T cells or Treg-deprived CD4^+^ T cells isolated from tolerant C57BL/6 mice were unable to efficiently respond to donor antigen stimulation in contrast to third-party antigens, this intrinsic functional unresponsiveness being sustained over the long term (>100 days posttransplant). Fourth, we observed that tolerated islet grafts harbor a significant proportion of CD4^+^ T-cells exhibiting a Foxp3^−^CD73^hi^FR4^hi^ phenotype, which has been correlated with an anergic state (16–18). Fifth, intragraft CD4^+^ T cells expressed high levels of the inhibitory receptor PD-1, and our recent report showed that blocking the PD-1/PD-L1 pathway at the time of CD3 Ab therapy or at day +100 posttransplant abolished immune tolerance and permanent islet graft survival (13). In addition, we have recently demonstrated that tolerated islets from CD3 Ab-treated C57BL/6 mice harbor CD8^+^ T cells deprived of proliferative, inflammatory, or cytotoxic capacities and that CD8^+^ T cell anergy was dependent on the presence of the alloantigens and on an *in situ* crosstalk between the PD-1/PD-L1 and TGFβ/TGFβRII pathways (13). This possibility of wholesale T-cell anergy can itself be sufficient to explain why PC61 and anti-CTLA-4 Abs failed to break the established tolerant state. *In vivo* demonstration of anergy to alloantigens has been poorly documented so far. One study in rat models showed that long-term survival of renal transplant after donor blood administration was associated with the inability of T cells to produce IL-2 and to proliferate and that treatment with recombinant IL-2 abrogated tolerance (27).

Our data are in line with a recent report documenting in type 1 diabetic (T1D) patients treated with humanized CD3 monoclonal Ab (teplizumab), the presence of dysfunctional CD8^+^ T cells qualified as “exhausted-like” and expressing the transcription factor Eomes, TIGIT, and KLRG-1 (28). In our islet transplant model, CD8^+^ T cells from CD3 Ab-treated tolerant mice also expressed Eomes and may well correspond to the subset described in T1D patients treated with CD3 Abs (13). In our model, we use the term “anergy” rather than “exhaustion” for both CD4^+^ and CD8^+^ T cells to define the unresponsive subsets in CD3 Ab-treated mice. In fact, although these cells did not respond to donor antigens, they maintained efficient responses to third-party antigens or polyclonal stimulus.

In conclusion, this study provides compelling evidence that Tregs play a substantive role in establishing a state of tolerance but that other mechanisms such as T cell anergy become more prominent for its maintenance and for long-term graft survival. This does not, of course, rule out a role for Foxp3^+^ T-cells in tolerance maintenance, as the level of their activity may, through homeostatic mechanisms, have been adjusted to that corresponding to residual effector function in the treated hosts (29, 30). These finding may be of importance for the implementation of Treg cell therapy in organ transplantation (31). One key point concerns Treg viability and persistence *in vivo*. In a hematopoietic stem cell transplantation clinical trial, infused Tregs were no longer detectable in the circulation after 2 weeks (32). It remains unclear whether the infused Tregs had become epigenetically destabilized and lost Foxp3 expression, had migrated to tissues, or had even died. Nonetheless, our data suggest that infused Tregs might enable sufficient immediate control of Teff function around the time of transplantation and immunotherapy, so permitting diverse intrinsic tolerogenic mechanisms to emerge and deliver the maintenance of a life-long tolerant state, with anergy being one such dominating mechanism.

## Author Contributions

AB and MB designed and performed experiments, analyzed the data, and wrote parts of the manuscript. TG and FV performed experiments and analyzed the data. HW and LC provided critical discussion and advices and reviewed the manuscript. SY designed and directed the study, analyzed the data, and wrote the manuscript.

## Conflict of Interest Statement

All authors declare that the research was conducted in the absence of any commercial or financial relationships that could be construed as a potential conflict of interest.
