# Supplementary material for: The Induction and Maintenance of Transplant Tolerance Engages Both Regulatory and Anergic CD4+ T cells
Source: Front Immunol. 2017 Mar 6;8:218. doi: 10.3389/fimmu.2017.00218 (PMC5337867; doi:10.3389/fimmu.2017.00218)
Supplement: Supplementary file 1 [file Table_1.DOCX]

**Table S1: Foxp3^+^ Treg elimination at the time of induction or maintenance of transplant tolerance: impact on graft survival.**

| **Transplanted organ/tissue** | **Combination** | **Therapy** | **Elimination of Tregs at the time of tolerance induction** | **Elimination of Tregs at the time of established tolerance** | **Ref.** |
| --- | --- | --- | --- | --- | --- |
| Skin | CB6F1 to TEA-Tg C57BL/6 | Anti-CD154 + DST | + | ND | (1) |
| Skin | BALB/c to DBA/2 | Rapamycin + IL-2 | + | ND | (2) |
| Skin | C57BL/6 to CBA | Anti-CD154 + DST | + | **-** | (3) |
| Skin | BALB/c to (CBAxB6hCD2) | anti-CD4/CD8/CD154 | ND | + (day 50) | (4) |
| Islets | BALB/c to C57BL/6 | Anti-CD45RB+rapamycin+IL-10 | + | - | (5; 6) |
| Islets | C57BL/6 to CBA | Anti-CD154 + DST | +/- | - | (3) |
| Islets | BALB/c to C57BL/6 | SA-FasL islets + rapamycin | + | + | (7) |
| Islets | C57BL/6 to BALB/c | Apoptotic spleen cells | + | ND | (8) |
| Islets | BALB/c to C57BL/6 | HLA-DQA1 + rapamycin | + | ND | (9) |
| Islets | BALB/c to C57BL/6 | CO + bilirubin | + | ND | (10) |
| Heart | BALB/c to C57BL/6 | DNT + rapamycin | + | ND | (11) |
| Heart | C57BL/6 to BALB/c | PVI of irradiated splenocytes | + | ND | (12) |
| Heart | BALB/c to C3H/HeJ | rapamycin | + | ND | (13) |
| Heart | BALB/c to C57BL/6 | HDACi + rapamycin | + | + (day 28) | (14) |
| Heart | BALB/c to C57BL/6 | Anti-CD154 | + | - | (15) |
|  |  |  |  |  |  |
| Skin | Male CBA to female A1(M)Tg-RAG^+^ | none | + | ND | (16) |
| Skin | (C57BL/6xbm12) to C57BL/6 | thymectomy | + | ND | (16) |
| Heart | B10.D2 to BALB/c | none | + | ND | (17) |
| Heart | BALB/c to B7^-/-^ B6 | none | + | ND | (18) |
| Liver | B10 to C3H | none | + | ND | (19) |
| Liver | DBA/2 to C3H | none | + | - | (20) |
| Kidney | BALB/c to IL-17^-/-^ B6 | none | + | ND | (21) |
| Kidney | BALB/c to Myd88^-/-^B6 | none | + | ND | (22) |
| Kidney | DBA/2 to B6.Foxp3^DTR^ | none | + | + | (23) |
|  |  |  |  |  |  |
| BM | Male to sub-irradiated female | none | + | - | (24) |
| BM + skin | BALB/c to irradiated B6 C57BL/6 | Anti-CD154 + DST | + chimerism  + skin | - chimerism  + skin | (25) |
| BM | B10.A to irradiated C57BL/6 | Anti-CD154 | - | ND | (26) |
| Splenocytes | C57BL/6 to irradiated B6C F1 | anti-CD154 + CTLA-4Ig | + | ND | (27) |
| BM + spleen | BDF1 to irradiated B6C3F1 | thymectomy | + | ND | (28) |
| BM + T cells | BALB/c to TBI B6.Foxp3^DTR^ | IL-33 | + | ND | (29) |
| BM + T cells | BALB/c to TBI B6.Foxp3^DTR^ | Cyclophosphamide | + | ND | (30) |
|  |  |  |  |  |  |
| EB | CBK to CBA/Ca (single MHC I) | none | + | ND | (31) |
| EB | 129 to C57BL/6 | CD3 Abs | + | - | (32) |
|  |  |  |  |  |  |
| Islets | Rat to mice | Anti-CD154 + rapamycin | + | - | (33) |
| Islets | Rat to mice | Anti-CD154 + rapamycin | + | - | (34) |
| Islets | Pig to mice | Anti-CD154 + anti-LFA-1 | ND | + | (35) |
| Islets | Human to μMT^-/-^ mice | Anti-CD45RB | + | +/- | (36) |

(+): graft rejection and abrogation of tolerance after Treg depletion, (-): no effect of Treg depletion on graft survival and tolerance, ND: not determined, EB: embryonic bodies, BM: bone marrow, DST: donor-specific transfusion, CO: carbon oxide, DNT: double negative T cells, PVI: portal vein injection, HDACi: histone deacetylase inhibitor.

**References**

1. Quezada SA, Bennett K, Blazar BR, et al. Analysis of the underlying cellular mechanisms of anti-CD154-induced graft tolerance: the interplay of clonal anergy and immune regulation. J Immunol. 2005;175:771-779

2. Pilon CB, Petillon S, Naserian S, et al. Administration of low doses of IL-2 combined to rapamycin promotes allogeneic skin graft survival in mice. Am J Transplant. 2014;14:2874-2882

3. Banuelos SJ, Markees TG, Phillips NE, et al. Regulation of skin and islet allograft survival in mice treated with costimulation blockade is mediated by different CD4+ cell subsets and different mechanisms. Transplantation. 2004;78:660-667

4. Kendal AR, Chen Y, Regateiro FS, et al. Sustained suppression by Foxp3+ regulatory T cells is vital for infectious transplantation tolerance. Journal of Experimental Medicine. 2011;208:2043-2053

5. Gagliani N, Gregori S, Jofra T, et al. Rapamycin combined with anti-CD45RB mAb and IL-10 or with G-CSF induces tolerance in a stringent mouse model of islet transplantation. PLoS One. 2011;6:e28434

6. Gagliani N, Jofra T, Valle A, et al. Transplant tolerance to pancreatic islets is initiated in the graft and sustained in the spleen. Am J Transplant. 2013;13:1963-1975

7. Yolcu ES, Zhao H, Bandura-Morgan L, et al. Pancreatic islets engineered with SA-FasL protein establish robust localized tolerance by inducing regulatory T cells in mice. J Immunol. 2011;187:5901-5909

8. Wu C, Zhang Y, Jiang Y, et al. Apoptotic cell administration enhances pancreatic islet engraftment by induction of regulatory T cells and tolerogenic dendritic cells. Cell Mol Immunol. 2013;10:393-402

9. Zang W, Lin M, Kalache S, et al. Inhibition of the alloimmune response through the generation of regulatory T cells by a MHC class II-derived peptide. J Immunol. 2008;181:7499-7506

10. Lee SS, Gao W, Mazzola S, et al. Heme oxygenase-1, carbon monoxide, and bilirubin induce tolerance in recipients toward islet allografts by modulating T regulatory cells. Faseb J. 2007;21:3450-3457

11. Zhang ZX, Lian D, Huang X, et al. Adoptive transfer of DNT cells induces long-term cardiac allograft survival and augments recipient CD4(+)Foxp3(+) Treg cell accumulation. Transpl Immunol. 2011;24:119-126

12. He F, Chen Z, Xu S, et al. Increased CD4+CD25+Foxp3+ regulatory T cells in tolerance induced by portal venous injection. Surgery. 2009;145:663-674

13. Urbanellis P, Shyu W, Khattar R, et al. The regulatory T cell effector molecule fibrinogen-like protein 2 is necessary for the development of rapamycin-induced tolerance to fully MHC-mismatched murine cardiac allografts. Immunology. 2015;144:91-106

14. Tao R, de Zoeten EF, Ozkaynak E, et al. Deacetylase inhibition promotes the generation and function of regulatory T cells. Nat Med. 2007;13:1299-1307

15. Jiang X, Sun W, Guo D, et al. Cardiac allograft acceptance induced by blockade of CD40-CD40L costimulation is dependent on CD4+CD25+ regulatory T cells. Surgery. 2011;149:336-346

16. Benghiat FS, Graca L, Braun MY, et al. Critical influence of natural regulatory CD25+ T cells on the fate of allografts in the absence of immunosuppression. Transplantation. 2005;79:648-654

17. Sho M, Yamada A, Najafian N, et al. Physiological mechanisms of regulating alloimmunity: cytokines, CTLA-4, CD25+ cells, and the alloreactive T cell clone size. J Immunol. 2002;169:3744-3751

18. Grazia TJ, Plenter RJ, Doan AN, et al. Spontaneous allograft tolerance in B7-deficient mice independent of preexisting endogenous CD4+CD25+ regulatory T-cells. Transplantation. 2007;83:1449-1458

19. Li W, Kuhr CS, Zheng XX, et al. New insights into mechanisms of spontaneous liver transplant tolerance: the role of Foxp3-expressing CD25+CD4+ regulatory T cells. Am J Transplant. 2008;8:1639-1651

20. Jiang X, Morita M, Sugioka A, et al. The importance of CD25+ CD4+ regulatory T cells in mouse hepatic allograft tolerance. Liver Transpl. 2006;12:1112-1118

21. Kwan T, Chadban SJ, Ma J, et al. IL-17 deficiency attenuates allograft injury and prolongs survival in a murine model of fully MHC-mismatched renal allograft transplantation. Am J Transplant. 2015;15:1555-1567

22. Wu H, Noordmans GA, O'Brien MR, et al. Absence of MyD88 signaling induces donor-specific kidney allograft tolerance. J Am Soc Nephrol. 2012;23:1701-1716

23. Miyajima M, Chase CM, Alessandrini A, et al. Early acceptance of renal allografts in mice is dependent on foxp3(+) cells. Am J Pathol. 2011;178:1635-1645

24. Weng L, Dyson J, Dazzi F. Low-intensity transplant regimens facilitate recruitment of donor-specific regulatory T cells that promote hematopoietic engraftment. Proc Natl Acad Sci U S A. 2007;104:8415-8420

25. Yamazaki M, Pearson T, Brehm MA, et al. Different mechanisms control peripheral and central tolerance in hematopoietic chimeric mice. Am J Transplant. 2007;7:1710-1721

26. Fehr T, Takeuchi Y, Kurtz J, et al. Early regulation of CD8 T cell alloreactivity by CD4+CD25- T cells in recipients of anti-CD154 antibody and allogeneic BMT is followed by rapid peripheral deletion of donor-reactive CD8+ T cells, precluding a role for sustained regulation. Eur J Immunol. 2005;35:2679-2690

27. Verbinnen B, Billiau AD, Vermeiren J, et al. Contribution of regulatory T cells and effector T cell deletion in tolerance induction by costimulation blockade. J Immunol. 2008;181:1034-1042

28. Inoue T, Ikegame K, Kaida K, et al. Host Foxp3+CD4+ Regulatory T Cells Act as a Negative Regulator of Dendritic Cells in the Peritransplantation Period. J Immunol. 2016;196:469-483

29. Matta BM, Reichenbach DK, Zhang X, et al. Peri-alloHCT IL-33 administration expands recipient T regulatory cells that protect mice against acute GVHD. Blood. 2016;

30. Ganguly S, Ross DB, Panoskaltsis-Mortari A, et al. Donor CD4+ Foxp3+ regulatory T cells are necessary for posttransplantation cyclophosphamide-mediated protection against GVHD in mice. Blood. 2014;124:2131-2141

31. Lui KO, Boyd AS, Cobbold SP, et al. A role for regulatory T cells in acceptance of ESC-derived tissues transplanted across an major histocompatibility complex barrier. Stem Cells. 2010;28:1905-1914

32. Calderon D, Prot M, You S, et al. Control of Immune Response to Allogeneic Embryonic Stem Cells by CD3 Antibody-Mediated Operational Tolerance Induction. Am J Transplant. 2016;16:454-467

33. Pan H, Lu HM, Hu WM, et al. Anti-CD25 mAb, anti-IL2 mAb, and IL2 block tolerance induction through anti-CD154 mAb and rapamycin in xenogeneic islet transplantation. Transplant Proc. 2007;39:3452-3454

34. Muller YD, Mai G, Morel P, et al. Anti-CD154 mAb and rapamycin induce T regulatory cell mediated tolerance in rat-to-mouse islet transplantation. PLoS One. 2010;5:e10352

35. Arefanian H, Tredget EB, Rajotte RV, et al. Short-term administrations of a combination of anti-LFA-1 and anti-CD154 monoclonal antibodies induce tolerance to neonatal porcine islet xenografts in mice. Diabetes. 2010;59:958-966

36. Zhao G, Moore DJ, Kim JI, et al. An immunosufficient murine model for the study of human islets. Xenotransplantation. 2014;21:567-573
